# Prognostic value of the Naples Prognostic Score in adult chronic obstructive pulmonary disease: NHANES 2005–2018

**DOI:** 10.3389/fnut.2024.1502266

**Published:** 2024-12-16

**Authors:** Yue Kang, Yingjian Tan, Yongxin Tie, Yujing Zhang, Chenyu Cui, Yuanyuan Zhao

**Affiliations:** ^1^Department of Pulmonary and Critical Care Medicine, Xinxiang Central Hospital, Xinxiang, China; ^2^Department of Pulmonary and Critical Care Medicine, The Fourth Clinical College of Xinxiang Medical University, Xinxiang, China; ^3^Department of Dermatology, Fuzhou First General Hospital, Fuzhou, China

**Keywords:** COPD, lung function, NHANES, Naples Prognostic Score, nutritional status

## Abstract

**Background:**

Previous studies have demonstrated that the Naples Prognostic Score (NPS) provides strategic direction in the prognosis of malignant illness. Nevertheless, its relationship with chronic obstructive pulmonary disease (COPD) remains underexplored. Therefore, additional research specifically focusing on the relationship between the Naples Prognostic Score and COPD is necessary to determine its widespread applicability.

**Objectives:**

The objective was to explore the relationship between the NPS and the susceptibility to developing COPD.

**Methods:**

A total of 15,184 participants were included in our research, and statistical analyses were performed after weighting. We used weighted logistic regression to assess whether the NPS and COPD had a relationship, as well as its association with lung function. Subgroup analysis was used to detect the interaction. In addition, Kaplan–Meier survival curves were generated, and adjusted hazard ratios of different causes of death were calculated using Cox regression.

**Results:**

Our investigation examined 15,184 participants’ data and then revealed a significant positive link between the NPS and COPD risk, while smoking has an interactive effect on it. A trend toward a decreasing prevalence of lung function indicators such as FEV1 [OR (95%CI), −339.70 (−456.53, −222.87)], FVC [OR (95%CI), −296.70 (−435.34, −158.06)], FEV1/FVC [OR (95%CI), −0.03 (−0.04, −0.02)], predicted FEV1 [OR (95%CI), −0.09 (−0.14, −0.04)], and predicted FVC [OR (95%CI), −0.08 (−0.15, −0.01)] was observed with increased NPS levels. Survival curves were drawn, and Cox regression analysis was conducted by dividing participants into group 0, group 1, and group 2 with NPS values of 0, 1–2, and 3–4, respectively. After assigning participants to groups 0, 1, and 2, with NPS values of 0, 1–2, and 3–4, respectively, the survival curves were created, and the Cox regression analysis was carried out. All-cause mortality [HR (95%CI), 1.68 (1.39–2.85)] and hypertension-related mortality [HR (95%CI), 67.64 (8.88–515.07)] of group 2 were significantly elevated.

**Conclusion:**

The findings of this study suggested that the NPS serves as a robust prognostic indicator of COPD.

## 1 Introduction

Chronic obstructive pulmonary disease (COPD) ranks among the most widespread pulmonary illnesses globally and constitutes a significant obstacle to public health. According to the 2021 Global Burden of Disease Study, it has become the third leading cause of age-standardized death around the globe, threatening long-term quality of life and placing a heavier socioeconomic load (1, 2). Considering its unusual symptoms, which are often confused with common upper respiratory infections, including cough and chest tightness, early-stage COPD is somewhat challenging to diagnose clinically. On the other hand, infections can lead to sudden flare-ups in medium- to late-stage patients as it progresses, which typically results in aggravation, sometimes even requiring mechanical ventilation (3). Nutritional imbalance, as it can lead to a decrease in overall muscle mass, is highly detrimental to lung function in COPD patients, thereby affecting the function of respiratory muscles and worsening the condition.

The Naples Prognostic Score (NPS) is a scoring system based on nutritional and inflammatory markers, initially used to assess the prognosis of patients with cancer (4). Recently, accumulating evidence suggests that it has also been applied to estimate the likelihood of developing other diseases.

The ratios of specific white blood cells such as neutrophils/lymphocytes (NLR) and lymphocytes/monocytes (LMR) have been shown in earlier research to function as diagnostic biomarkers to evaluate aggravations in COPD patients (5). In addition, by influencing immunomodulation and pharmacokinetics, nutritional indicators such as albumin indirectly affect patient outcomes and prognosis (6). This study explored the relationship of NPS with COPD incidence and prognosis using the NHANES data from 2005 to 2018.

## 2 Materials and methods

### 2.1 Data processing

The NHANES is a biennial survey that collects laboratory tests, examinations, demographic information, and questionnaires to deliver insights into the health and dietary conditions of Americans. To reliably and representatively reflect the non-institutionalized U.S. population, it uses a sophisticated multistage stratified sampling method, making the data suitable for the evaluation of nutrition and health (7). It was conducted with ethical approval from the National Center for Health Statistics (NCHS). Data from seven NHANES cycles were gathered for this study (2005–2018). We included adults over the age of 20, except pregnant women and people with extreme nutritional consumption (men over 4,200 or under 800 kcal/day; women over 3,500 or under 500 kcal/day) (8). For missing data on exposure factors (NPS assessment) and outcome factors (COPD assessment) and missing covariate data of age, sex, and ethnicity, deletion was performed. Multiple imputation was used to fill in the other missing covariate data.

### 2.2 NPS assessment

The NPS is determined by summing up the scores of four key parameters: NLR ≥ 2.96, albumin level < 40 g/L, LMR ≤ 4.44, and total cholesterol level ≤ 180 mg/dL, each receive one point, otherwise receive zero points (9). Adding up the scores of the four parameters, then diving them into three groups which are group 0 (NPS of 0), group 1 (NPS of 1 to 2), and group 2 (NPS of 3 to 4).

### 2.3 COPD assessment

According to the Global Initiative for Chronic Obstructive Lung Disease (GOLD) COPD is defined as: (1) The ratio of FEV1/FVC < 0.7 following bronchodilator usage. (2) Age over 40 years old with a history of smoking, emphysema or chronic bronchitis (Health Conditions questionnaire: MCQ160G, MCQ160K, MCQ160P, MCQ170K, MCQ160O); The cited references remain unchanged (10–12).

### 2.4 Mortality assessment

For this study, the survey data were correlated with the National Death Index^1^ to obtain mortality data. The follow-up period commenced from the NHANES interview date and continued until either 31 December 2019 or the participant’s date of death.

### 2.5 Lung function test

This study collected lung function data (including FEV1, FEV1, and FEV1/FVC) prior to the use of bronchodilators. For quality control purposes, only data rated as A or B quality were included. Then, the corresponding expected values were calculated based on the Hankinson equation (13).

### 2.6 Covariates

Building upon previous research, this study incorporated several covariates to control potential confounding variables. These included poverty-to-income ratio (14), which is calculated by dividing household income by the federal poverty line income and classified into the following categories: ≤1.0, 1.1–3.0, and > 3.0; age (in years); education level (below high school, high school, or above high school); sex (male or female); smoking status (never, former, or current); and ethnicity (Mexican American, other Hispanic, non-Hispanic white, non-Hispanic black, or other) (14, 15).

### 2.7 Statistical analysis

Following the CDC guidelines^2^, statistical analyses were performed with sample weights taken into account. Appropriate weights were also assigned to each individual to be representative of the American population. Following the “least common denominator” principle, the weight calculation formula is 1/7 * WTMEC2YR. Baseline characteristics of enrolled patients were described according to the NPS grouping and mortality status. For comparison, weighted ANOVA was used for continuous variables (mean ± SD), and weighted chi-square tests were used for categorical variables (percentages). Weighted logistic regression analysis was used to investigate the association between NPS and COPD incidence, as well as NPS and various lung function indicators in participants with COPD. Using the Kaplan–Meier to create survival curves based on the NPS grouping, a log-rank test was performed. Adjusted hazard ratios (HRs) and 95% confidence intervals (CIs) of different causes of death were calculated using Cox regression. Both the unadjusted and adjusted models were used. Model 1 was adjusted for age, ethnicity, and sex. Model 2 was adjusted for all relevant covariates. Stratified logistic regression and interaction tests were used to confirm the reliability of the results. Spearman’s correlation analysis was used to evaluate the correlation coefficients between the NPS and its components. ROC curves were used to compare the predictive value of NPS and its parameters for all-cause mortality in COPD patients. R software and EmpowerStats 4.2 were used for all statistical analyses and visualizations. A *p*-value of <0.05 on both sides was recognized as statistically significant.

## 3 Results

### 3.1 Baseline characteristics

**Figure 1:**
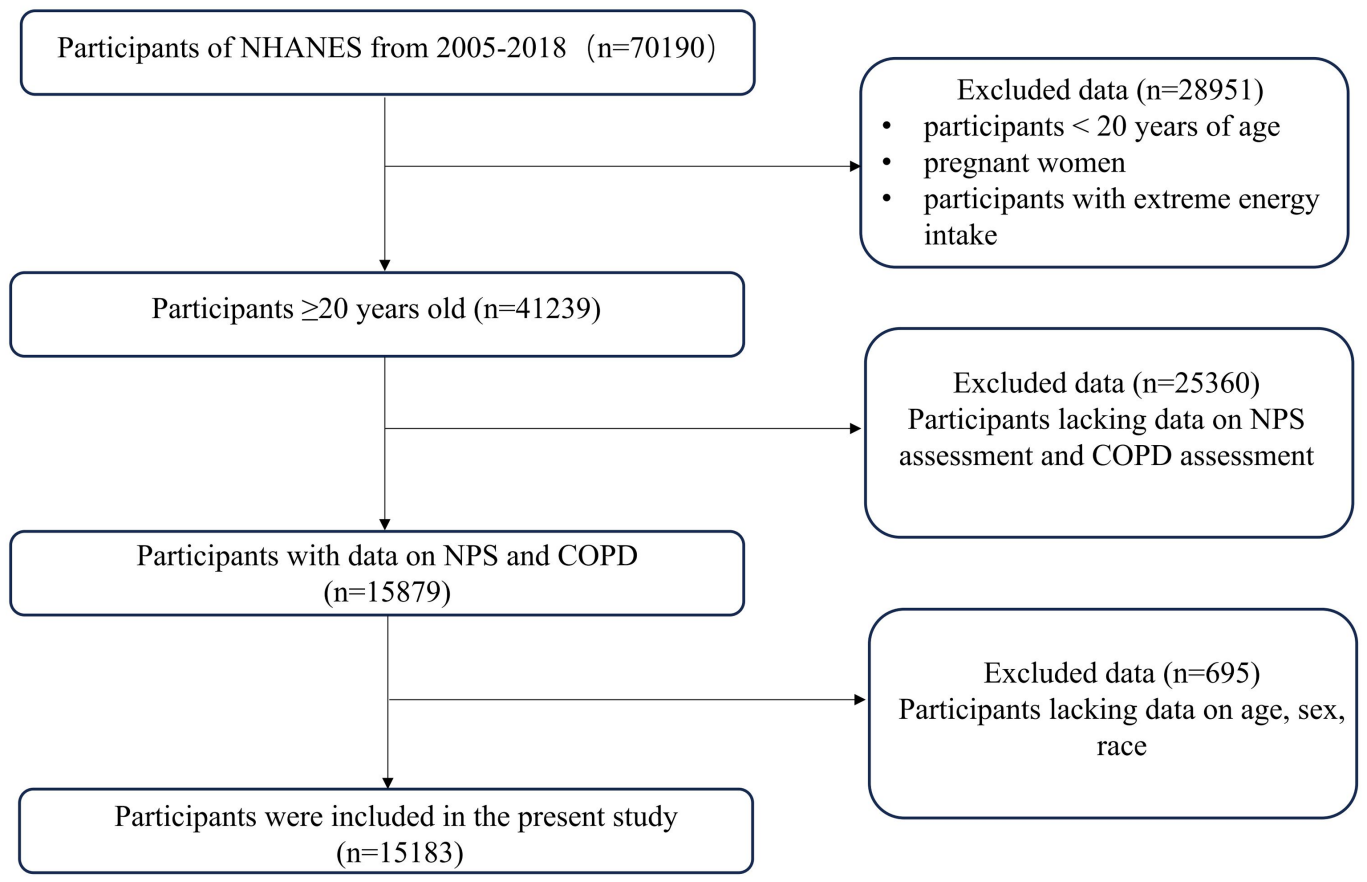
Admission flowchart.

A total of 15,184 participants were enrolled in this study, of which 1,466 (9.65%) participants were diagnosed with COPD. Individuals younger than 20 years old (*n* = 28,951) and those lacking NPS or COPD assessment data (*n* = 25,360) were excluded from the study. Moreover, participants lacking covariate data (*n* = 695) were excluded from the final analysis, as shown in Figure 1. The weighted distribution of participant demographics and other covariates by NPS groupings is shown in Table 1. Group 0, group 1, and group 2 represented 16.52, 69.93, and 13.55% of the participants, respectively. The average age of enrolled participants was 51.57 ± 17.42 years, with 59.17% being male, and the majority identified as non-Hispanic white, accounting for 50.43%. Among the three groups, significant differences were observed regarding sex, age, ethnicity, smoking status, NLR levels, LMR levels, and a medical history that included asthma, emphysema, chronic bronchitis, and COPD. The participants of group 2 were generally older, predominantly non-Hispanic white, also had lower income, and were more likely to have COPD and smoking history. Table 2 shows that after weighting, there were 10.53% all-cause deaths among participants. Compared to survivors, deceased participants were likely to be senior, non-Hispanic white men with a smoking habit, typically with lower education levels and income, and they had higher NPSs.

**Table 1:** Characteristics of different NPS groups.

Characteristics	Naples prognostic score			*p*-value
	Group 0	Group 1	Group 2	
Age, years	47.87 ± 14.95	49.02 ± 16.73	53.55 ± 18.51	<0.001
Sex (Female), %	1,326 (52.26)	4,242 (41.07)	1,010 (43.54)	<0.001
Ethnicity, %				0.001
Mexican American	273 (10.75)	828 (8.02)	175 (7.54)	
Other Hispanic	139 (5.46)	578 (5.60)	132 (5.70)	
Non-Hispanic white	1,588 (62.61)	7,009 (67.86)	1,576 (67.94)	
Non-Hispanic black	352 (13.87)	1,256 (12.16)	300 (12.94)	
Other	185 (7.31)	657 (6.36)	137 (5.89)	
Educational background, %				0.079
Below high school	537 (21.16)	2,190 (21.20)	550 (23.73)	
High school	710 (27.97)	2,735 (26.48)	649 (27.99)	
Above high school	1,291 (50.87)	5,404 (52.32)	1,119 (48.27)	
PIR, %				0.019
≤1.0	2,202 (86.80)	8,914 (86.31)	2081 (89.75)	
1.1–3.0	122 (4.79)	609 (5.90)	115 (4.98)	
>3.0	213 (8.41)	805 (7.79)	122 (5.26)	
Smoking status, %				<0.001
Never	1,004 (39.57)	3,928 (38.03)	818 (35.26)	
Former	291 (11.46)	820 (7.94)	153 (6.61)	
Current	1,243 (48.98)	5,581 (54.04)	1,348 (58.13)	
NLR	1.55 [1.21, 1.93]	2.00 [1.56, 2.55]	3.36 [2.86, 4.15]	<0.001
LMR	5.50 [4.83, 6.25]	3.67 [3.00, 4.33]	2.71 [2.13, 3.33]	<0.001
Asthma, %	407 (16.04)	1,658 (16.05)	449 (19.38)	0.013
Bronchitis, %	253 (9.99)	842 (8.15)	247 (10.63)	0.011
Emphysema, %	86 (3.38)	377 (3.65)	134 (5.79)	0.001
COPD, %	272 (10.71)	887 (8.59)	271 (11.70)	0.003

**Table 2:** Characteristics of different survival statuses.

Characteristics	All-cause mortality		*p*-value
	No	Yes	
Age (years)	47.87 ± 16.14	62.88 ± 16.16	<0.001
Sex (Female), %	5,697 (43.88)	831 (37.73)	<0.001
Ethnicity, %			<0.001
Mexican American	1,115 (8.59)	150 (6.79)	
Other Hispanic	754 (5.81)	82 (3.72)	
Non-Hispanic white	8,607 (66.30)	1,610 (73.11)	
Non-Hispanic black	1,640 (12.63)	260 (11.81)	
Other	866 (6.67)	101 (4.58)	
Education level, %			<0.001
Below high school	2,673 (20.59)	653 (29.64)	
High school	3,512 (27.05)	565 (25.65)	
Above high school	6,797 (52.36)	985 (44.71)	
PIR, %			<0.001
≤1.0	11,394 (87.77)	1739 (78.99)	
1.1–3.0	627 (4.83)	268 (12.15)	
>3.0	961 (7.40)	195 (8.86)	
Smoking status, %			<0.001
Never	4,946 (38.10)	796 (36.14)	
Former	1,129 (8.70)	114 (5.16)	
Current	6,906 (53.20)	1,292 (58.70)	
NLR	2.00 [1.52, 2.64]	2.23 [1.62, 3.16]	<0.001
LMR	3.80 [3.00, 4.80]	3.43 [2.50, 4.50]	<0.001
Asthma, %	2,133 (16.43)	373 (16.95)	0.63
Bronchitis, %	1,083 (8.34)	279 (12.68)	<0.001
Emphysema, %	378 (2.91)	271 (12.31)	<0.001
COPD, %	1,062 (8.18)	429 (19.46)	<0.001

### 3.2 Relationship between the NPS and COPD

**Figure 2:**
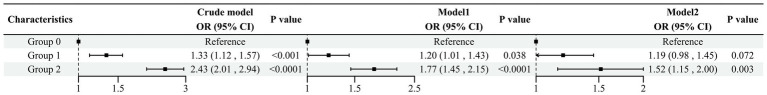
Association between the NPS and COPD Model 1 adjusted for sex, ethnicity, and age. Model 2 adjusted for sex, race, age, PIR, educational background, TC, ALB, neutrophils, lymphocytes, monocytes, and smoking status.

The weighted logistic regression analysis demonstrated the relationship between the NPS and COPD, with group 0 serving as the reference in Figure 2. The crude model showed a positive association between NPS and COPD prevalence [OR (95%CI), 2.43 (2.01–2.94)]. In Model 2, after adjusting for age, PIR, educational background, ethnicity, TC, ALB, sex, neutrophils, lymphocytes, monocytes, and smoking status, the risk of COPD increased by 52% in group 2 compared to group 0. The trend analysis in Model 2 showed that the risk of COPD increased in tandem with an increase in the NPSs (*p* = 0.0007).

### 3.3 Relationship between NPS and mortality in COPD

**Figure 3:**
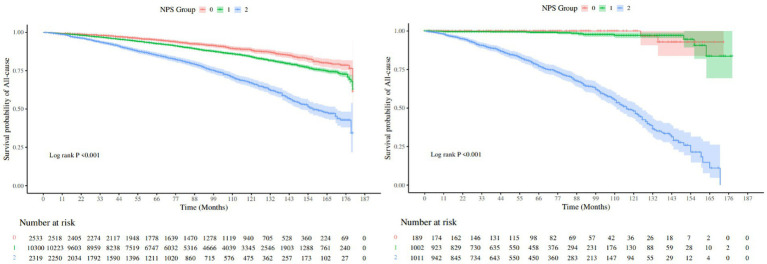
KM survival curves.

We used the Kaplan–Meier method to plot the survival curve and to evaluate the prognostic value of NPS in COPD patients, as shown in Figure 3. The results demonstrated that group 2 had the highest risk for both all-cause mortality and hypertension-related mortality compared to other groups (log-rank, *p* < 0.001). After adjusting for multiple variables, all-cause mortality [HR (95%CI), 1.68 (1.39–2.85)] and hypertension-related mortality [HR (95%CI), 67.64 (8.88–515.07)] of group 2 were significantly elevated in comparison to group 0, as shown in Table 3. The trend analysis demonstrated that as the NPS increased, both all-cause mortality and hypertension-related mortality rose accordingly.

**Table 3:** Association between NPS and mortality.

	Naples prognostic score			*P* for trend
	Group 0 OR (95%CI)	Group 1 OR (95%CI)	Group 2 OR (95%CI)	
All-cause mortality				<0.0001
Crude	References	1.39 (1.22–1.59) <0.001	3.22 (2.78–3.73) < 0.0001	
Model 1	References	1.22 (1.07–1.39) 0.004	2.04 (1.76–2.37) < 0.001	
Model 2	Reference	1.17 (1.01–1.35) 0.037	1.68 (1.39–2.02) < 0.001	
Hypertension				
Crude	Reference	1.67 (0.38–7.32) 0.494	40.06 (9.97–160.98) < 0.001	<0.0001
Model 1	Reference	1.98 (0.45–8.65) 0.366	45.46 (11.30–182.85) < 0.001	
Model 2	Reference	4.07 (0.52–31.67) 0.180	67.64 (8.88–515.07) < 0.001	

Model 1 adjusted for sex, ethnicity, and age.

Model 2 adjusted for sex, ethnicity, age, PIR, educational background, TC, ALB, neutrophils, lymphocytes, monocytes, and smoking status.

### 3.4 Subgroup analysis

**Figure 4:**
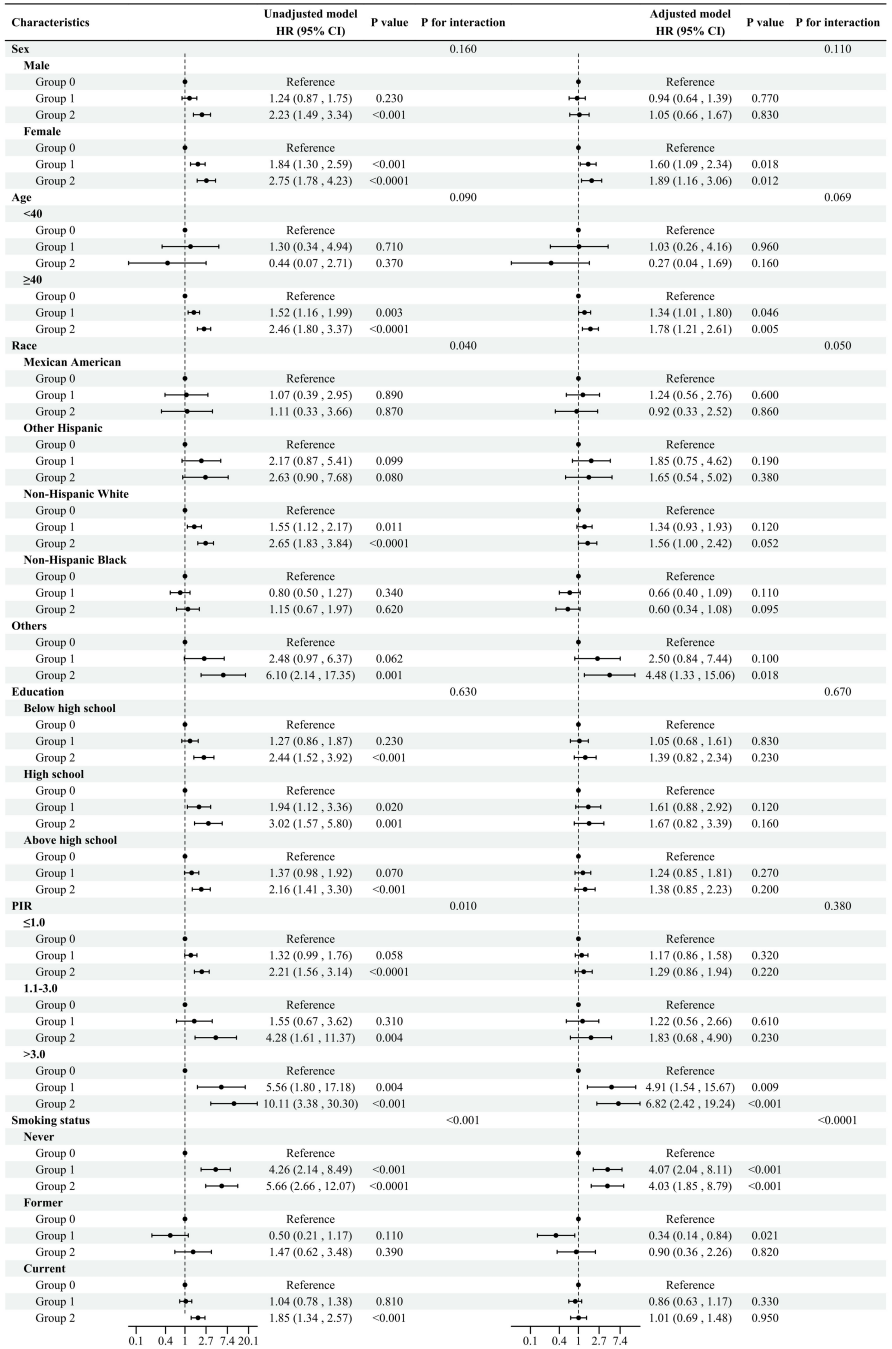
Subgroup analysis Adjusted model adjusted for sex, ethnicity, age, PIR, educational background, TC, ALB, neutrophils, lymphocytes, monocytes, and smoking status. All the models are not adjusted for the variable itself in each stratification.

In Figure 4, we further explored the association between the NPS and COPD risk by conducting subgroup analyses based on age, sex, ethnicity, educational background, PIR, and whether they smoked. In the majority of subgroups, the risk of COPD increased with elevated NPS levels, and the same results were observed in the adjusted models. Notably, in the adjusted model, in terms of smoking, the odds ratios (ORs, 95%CI) of group 2 for the risk of COPD are as follows: never smokers 4.03 (1.85, 8.79), former smokers 0.90 (0.36, 2.26), and current smokers 1.01 (0.69, 1.48), and the interaction analysis showed *p* < 0.05. The results indicated that in the adjusted model, smoking history influenced the relationship between the NPS and COPD prevalence. Other covariates, such as ethnicity, PIR, and education, did not have a significant impact on COPD incidence.

### 3.5 Relationship between lung function and NPS

With group 0 serving as the reference, after adjusting for multiple variables, group 2 was negatively correlated with FEV1, predicted FEV1, FVC, predicted FVC, and FEV1/FVC, and the odds ratios (OR, 95%CI) were −339.70 (−456.53, −222.87), −296.70 (435.34, −158.06), −0.03 (−0.04, −0.02), −0.09 (−0.14, −0.04), and −0.08 (−0.15, −0.01), respectively. These results were statistically significant, as shown in Table 4. The trend analysis results indicated that with increasing NPS, FEV1, FVC, FEV1/FVC, and predicted FEV1, all showed a downward trend (*p* < 0.05).

**Table 4:** Association between NPS and lung function.

Lung function test	Naples prognostic score			*P* for trend
	Group 0 OR (95%CI)	Group 1 OR (95%CI)	Group 2 OR (95%CI)	
FEV1	Reference	31.68 (−39.93, 103.29)	−339.70 (−456.53, −222.87)	0.003
FVC	Reference	60.77 (−24.21,145.75)	−296.70 (−435.34, −158.06)	<0.001
FEV1/FVC	Reference	−0.003 (−0.01, 0.0005)	−0.031 (−0.04, −0.02)	<0.001
FEV1 predicted	Reference	0.02 (−0.01, 0.05)	−0.09 (−0.14, −0.04)	0.03
FVC predicted	Reference	0.03 (−0.01,0.07)	−0.08 (−0.15, −0.01)	0.23
FEV1/FVC% predicted	Reference	0 (−0.001, 0.001)	0 (−0.001, 0.002)	0.64

### 3.6 Prognostic value of the NPS

We investigated the correlation between the NPS and its components and observed a significant positive correlation between NLR and NPS (*r* = 0.51), as shown in Appendix Figure 1A. In terms of prognostic value, we compared the NPS with its components and found that the area under the curve of the NPS was the largest (AUC = 0.701), which showed that the NPS is the most effective predictor of all-cause mortality in adults with COPD, as shown in Appendix Figure 1B and Appendix Table 1.

## 4 Discussion

This large-scale study, which included 15,184 participants from the NHANES database, comprehensively examined the relationship of NPS with both the risk and prognosis of COPD. Our findings revealed that higher NPSs and the occurrence of COPD were found to be significantly correlated, and this relationship remained stable after adjustment. Likewise, higher NPSs were related to higher all-cause and hypertension-related mortality rates in COPD patients. Logistic regression analysis showed significant negative correlations between NPS and key pulmonary function parameters. Furthermore, trend analysis revealed a decline in lung function as NPS levels increased. ROC curves showed that NPS had the strongest prognostic value compared to its components.

Chronic inflammation is broadly acknowledged as a primary mechanism in the onset of COPD (10). Previous research primarily emphasized specific inflammatory or nutritional markers in COPD patients, such as the NLR and LMR, both of which are associated with the pathogenesis and prognosis of the disease. These markers reflect the intricate balance between inflammation and immune response, making them valuable indicators for assessing disease progression and clinical outcomes conditions (14, 16–18). Research has shown that malnutrition affects up to 74.4% of elderly patients with COPD. This condition significantly impairs immune function, diminishes muscle strength, and reduces respiratory capacity, all of which contribute to accelerated disease progression. Consequently, malnutrition is connected to an elevated risk of complications, extended hospitalizations, and increased mortality rates (19, 20).

The NPS, as a composite score, reflects systemic inflammation and nutritional status by incorporating four key biomarkers—ALB, TC, NLR, and LMR. It was originally designed to evaluate prognosis in cancer patients (21–26). Recent research has indicated that low albumin levels often signify malnutrition, which can impair immune response, reduce lung muscle strength, and diminish respiratory function, thereby accelerating COPD progression (19, 20, 27). Concurrently, elevated inflammatory markers such as NLR and PLR represent a sustained systemic inflammatory state, which exacerbates lung damage and contributes to immune system dysregulation, worsening patient outcomes (18, 28). Moreover, recent studies have highlighted the role of lipid metabolism disorders in COPD pathology, with a notable relationship between increased cholesterol levels, deteriorating lung function, and heightened oxidative stress (29–33). Therefore, by integrating these critical factors, the NPS serves as an effective tool for predicting disease progression and adverse outcomes in COPD patients.

While markers such as NLR and PLR may indicate systemic inflammation, they do not necessarily account for all factors that contribute to disease progression in COPD patients. In addition, the role of lipid metabolism disorders in COPD pathology does not solely determine disease progression and adverse outcomes as other variables must also be considered in predicting patient outcomes.

A previous study by Zhu (34), also based on the NHANES data, demonstrated a notable link between higher NPSs and asthma incidence. Another study by Wu (35) reported that elevated NPSs were linked to increased occurrences of respiratory symptoms (e.g., cough, wheezing, and dyspnea) and an increased predisposition to obstructive and restrictive lung function abnormalities. Another study demonstrated a strong positive association between the NPS and both overall mortality risk and cause-specific mortality risk among cancer patients (26). Our findings align with previous studies, reaffirming the NPS as a reliable prognostic marker. In addition, subgroup analysis indicated that the link between the NPS and COPD incidence was more pronounced in individuals with smoking habits after adjusting for confounders.

From a clinical perspective, the findings of this study demonstrated significant practical applicability. The four components of the NPS encompass routine laboratory tests, including complete blood count, blood lipids, and blood biochemistry, which are commonly available at the initiation of comprehensive assessments upon patient admission. The NPS is calculated based on these test results to stratify patients accordingly. A higher NPS is often associated with a poorer prognosis and serves as a prompt for clinicians to consider early nutritional support, such as intravenous albumin. In addition, it underscores the need for timely infection control interventions, including the collection of sputum samples for microbiological analysis and the escalation of antimicrobial therapy when necessary. Early respiratory support, such as high-flow oxygen therapy or non-invasive ventilation, may also be warranted. Throughout the treatment course, dynamic monitoring of NPS variations can provide valuable insights into the patient’s response to therapy and overall prognosis. Furthermore, COPD patients, who frequently experience chronic hypoxia and pulmonary vascular remodeling, are at increased risk for cardiovascular complications, including cor pulmonale and pulmonary embolism. Incorporating the measurement of cardiovascular biomarkers, such as B-type natriuretic peptide, troponin, and D-dimer, in patients with high NPS can facilitate early intervention and potentially improve clinical outcomes.

Despite these significant findings, several limitations should be acknowledged. First, due to the reliance on the NHANES database, which primarily reflects the U.S. population, cross-national validation was not feasible. Second, although the NHANES uses standardized controls to ensure data reliability and completeness, the retrospective cohort design makes it difficult to completely eliminate confounding bias and establish causality. Third, the reliance on self-reported COPD diagnoses carries the potential for recall bias, which could impact the precision of the results. Therefore, future research could consider multicenter studies across diverse populations to evaluate the external validity and generalizability of the NPS in different ethnic groups. Moreover, conducting longitudinal studies is essential for strengthening causal inferences and evaluating the predictive value of NPS in relation to long-term clinical outcomes.

In conclusion, our study showed that higher NPSs are strongly linked to COPD onset and increased all-cause mortality, including hypertension-related deaths, in COPD patients. Based on routine laboratory tests, the NPS offers a practical, cost-effective tool for the early identification of high-risk COPD patients and has significant prognostic value. Furthermore, combining NPS with cardiovascular biomarkers can enhance the early diagnosis and management of patients with cardiac comorbidities. Given its clinical relevance, we recommend incorporating the NPS into routine practice for risk stratification in COPD management.

## Data Availability

Publicly available datasets were analyzed in this study. This data can be found here: https://wwwn.cdc.gov/nchs/nhanes/Default.aspx.

## Footnotes

^1^ https://www.cdc.gov/

^2^ https://wwwn.cdc.gov/nchs/nhanes/tutorials/default.aspx

## Supplementary material

APPENDIX FIGURE 1A Spearman’s correlation analysis among NPS and its components.

APPENDIX FIGURE 1B ROC curves of NPS and its components in predicting all-cause mortality in COPD patients.

## References

[1] GBD 2021 Risk Factors Collaborators. Global burden and strength of evidence for 88 risk factors in 204 countries and 811 subnational locations, 1990-2021: a systematic analysis for the global burden of disease study 2021. Lancet. (2024) 403:2162–203. doi: 10.1016/S0140-6736(24)00933-4, PMID: 38762324 PMC11120204

[2] Chen S Kuhn M Prettner K Yu F Yang T Bärnighausen T. The global economic burden of chronic obstructive pulmonary disease for 204 countries and territories in 2020-50: a health-augmented macroeconomic modelling study. Lancet Glob Health. (2023) 11:e1183–93. doi: 10.1016/S2214-109X(23)00217-6, PMID: 37474226 PMC10369014

[3] Stephenson H Roberts M Klimkeit E Smith T. Uncovering undernutrition in chronic obstructive pulmonary disease: beyond body mass index. Respir Med. (2022) 205:107026. doi: 10.1016/j.rmed.2022.107026, PMID: 36395571

[4] Galizia G Lieto E Auricchio A Cardella F Mabilia A Podzemny V. Naples prognostic score, based on nutritional and inflammatory status, is an independent predictor of long-term outcome in patients undergoing surgery for colorectal Cancer. Dis Colon Rectum. (2017) 60:1273–84. doi: 10.1097/DCR.0000000000000961, PMID: 29112563

[5] Cai C Zeng W Wang H Ren S. Neutrophil-to-lymphocyte ratio (NLR), platelet-to-lymphocyte ratio (PLR) and monocyte-to-lymphocyte ratio (MLR) as biomarkers in diagnosis evaluation of acute exacerbation of chronic obstructive pulmonary disease: a retrospective, observational study. Int J Chron Obstruct Pulmon Dis. (2024) 19:933–43. doi: 10.2147/COPD.S452444, PMID: 38646605 PMC11027921

[6] Ling M Huiyin L Shanglin C Haiming L Zhanyi D Shuchun W. Relationship between human serum albumin and in-hospital mortality in critical care patients with chronic obstructive pulmonary disease. Front Med. (2023) 10:1109910. doi: 10.3389/fmed.2023.1109910, PMID: 37181348 PMC10174316

[7] Vogtmann E Chaturvedi AK Blaser MJ Bokulich NA Caporaso JG Gillison ML. Representative oral microbiome data for the US population: the National Health and nutrition examination survey. Lancet Microbe. (2023) 4:e60–1. doi: 10.1016/S2666-5247(22)00333-0, PMID: 36455567

[8] Yang T Yi J He Y Zhang J Li X Ke S. Associations of dietary fats with all-cause mortality and cardiovascular disease mortality among patients with Cardiometabolic disease. Nutrients. (2022) 14:3608. doi: 10.3390/nu14173608, PMID: 36079863 PMC9460477

[9] Arnett DK Blumenthal RS Albert MA Buroker AB Goldberger ZD Hahn EJ. 2019 ACC/AHA guideline on the primary prevention of cardiovascular disease: a report of the American College of Cardiology/American Heart Association task force on clinical practice guidelines. Circulation. (2019) 140:e596–646. doi: 10.1161/CIR.0000000000000678, PMID: 30879355 PMC7734661

[10] Global Initiative for Chronic Obstructive Lung Disease. Global strategy for prevention, diagnosis and management of COPD: 2024 report. (2024). Available at: https://goldcopd.org/2024-gold-report/.

[11] Lin ZF Lin HW Liao WZ Huang ZM Liao XY Wang YY. The association between dietary magnesium intake with chronic obstructive pulmonary disease and lung function in US population: a cross-sectional study. Biol Trace Elem Res. (2024) 202:3062–72. doi: 10.1007/s12011-024-04073-z, PMID: 38273185

[12] Lan CC Su WL Yang MC Chen SY Wu YK. Predictive role of neutrophil-percentage-to-albumin, neutrophil-to-lymphocyte and eosinophil-to-lymphocyte ratios for mortality in patients with COPD: evidence from NHANES 2011-2018. Respirology. (2023) 28:1136–46. doi: 10.1111/resp.14589, PMID: 37655985

[13] Miller MR Hankinson J Brusasco V Burgos F Casaburi R Coates A. Standardisation of spirometry. Eur Respir J. (2005) 26:319–38. doi: 10.1183/09031936.05.00034805 16055882

[14] Paliogiannis P Fois AG Sotgia S Mangoni AA Zinellu E Pirina P. Neutrophil to lymphocyte ratio and clinical outcomes in COPD: recent evidence and future perspectives. Eur Respir Rev. (2018) 27:170113. doi: 10.1183/16000617.0113-2017 29436405 PMC9488932

[15] Qiu Z Chen X Geng T Wan Z Lu Q Li L. Associations of serum carotenoids with risk of cardiovascular mortality among individuals with type 2 diabetes: results from NHANES. Diabetes Care. (2022) 45:1453–61. doi: 10.2337/dc21-2371, PMID: 35503926

[16] Rahimirad S Ghaffary MR Rahimirad MH Rashidi F. Association between admission neutrophil to lymphocyte ratio and outcomes in patients with acute exacerbation of chronic obstructive pulmonary disease. Tuberk Toraks. (2017) 65:25–31. doi: 10.5578/tt.27626, PMID: 28621246

[17] El-Gazzar AG Kamel MH Elbahnasy OKM El-Naggar ME. Prognostic value of platelet and neutrophil to lymphocyte ratio in COPD patients. Expert Rev Respir Med. (2020) 14:111–6. doi: 10.1080/17476348.2019.1675517, PMID: 31577911

[18] Mirna M Schmutzler L Topf A Hoppe UC Lichtenauer M. Neutrophil-to-lymphocyte ratio and monocyte-to-lymphocyte ratio predict length of hospital stay in myocarditis. Sci Rep. (2021) 11:18101. doi: 10.1038/s41598-021-97678-6, PMID: 34518607 PMC8438016

[19] Perrot L Greil A Boirie Y Farigon N Mulliez A Costes F. Prevalence of sarcopenia and malnutrition during acute exacerbation of COPD and after 6 months recovery. Eur J Clin Nutr. (2020) 74:1556–64. doi: 10.1038/s41430-020-0623-6, PMID: 32296123

[20] Cunha AIL Veronese N de Melo BS Ricci NA. Frailty as a predictor of adverse outcomes in hospitalized older adults: a systematic review and meta-analysis. Ageing Res Rev. (2019) 56:100960. doi: 10.1016/j.arr.2019.100960 31518686

[21] Gu J Deng S Jiang Z Mao F Xue Y Qin L. Modified Naples prognostic score for evaluating the prognosis of patients with obstructive colorectal cancer. BMC Cancer. (2023) 23:941. doi: 10.1186/s12885-023-11435-8, PMID: 37798689 PMC10557152

[22] Zhu N Lin S Cao C. A novel prognostic prediction indicator in patients with acute pulmonary embolism: Naples prognostic score. Thromb J. (2023) 21:114. doi: 10.1186/s12959-023-00554-8, PMID: 37932805 PMC10629175

[23] Xu B Zhu J Wang R Pang X Wang X Lian J. Clinical implications of Naples prognostic score for patients with resected cholangiocarcinoma: a real-world experience. J Inflamm Res. (2024) 17:655–67. doi: 10.2147/JIR.S446735, PMID: 38328562 PMC10849881

[24] Song C Yu D Li Y Liu M Zhang H He J. Predictive value of the Naples prognostic score on postoperative delirium in the elderly with gastrointestinal tumors: a retrospective cohort study. BMC Geriatr. (2024) 24:535. doi: 10.1186/s12877-024-05113-y, PMID: 38902614 PMC11188257

[25] Guo H Wang T. Predictive role of Naples prognostic score for survival in esophageal cancer: a meta-analysis. Medicine. (2024) 103:e38160. doi: 10.1097/MD.0000000000038160, PMID: 38787991 PMC11124694

[26] Liang C Zhang C Song J Yan L Xiao Y Cheng N. The Naples prognostic score serves as a predictor and prognostic indicator for cancer survivors in the community. BMC Cancer. (2024) 24:696. doi: 10.1186/s12885-024-12448-7, PMID: 38844884 PMC11157788

[27] Feng Y Xu W Tang S Ye Z Fang P Abdullah G. Inflammation, nutrition, and biological aging: the prognostic role of Naples prognostic score in nonalcoholic fatty liver disease outcomes. Diabetes Res Clin Pract. (2024) 213:111749. doi: 10.1016/j.diabres.2024.111749, PMID: 38906332

[28] Dang P Wang F Yu H. Prognostic potential of neutrophil-to-lymphocyte ratio, platelet to lymphocyte ratio, and monocyte to lymphocyte ratio in acute myocardial infarction patients combined with chronic obstructive pulmonary disease. Front Cardiovasc Med. (2024) 11:1401634. doi: 10.3389/fcvm.2024.1401634, PMID: 39070559 PMC11272454

[29] McQueen MJ Hawken S Wang X Ounpuu S Sniderman A Probstfield J. Lipids, lipoproteins, and apolipoproteins as risk markers of myocardial infarction in 52 countries (the INTERHEART study): a case-control study. Lancet. (2008) 372:224–33. doi: 10.1016/S0140-6736(08)61076-4, PMID: 18640459

[30] Eriksson B Lindberg A Müllerova H Rönmark E Lundbäck B. Association of heart diseases with COPD and restrictive lung function--results from a population survey. Respir Med. (2013) 107:98–106. doi: 10.1016/j.rmed.2012.09.011, PMID: 23127573

[31] Wen X Wu X Deng Z Wu F Yang H Xiao S. The nonlinear relationship between high-density lipoprotein and changes in pulmonary structure function and pulmonary function in COPD patients in China. Int J Chron Obstruct Pulmon Dis. (2024) 19:1801–12. doi: 10.2147/COPD.S467976, PMID: 39129965 PMC11316472

[32] Shen Y Yang T Guo S Li X Chen L Wang T. Increased serum ox-LDL levels correlated with lung function, inflammation, and oxidative stress in COPD. Mediat Inflamm. (2013) 2013:972347:1–5. doi: 10.1155/2013/972347 PMC3774040 24078777

[33] Nadeem A Raj HG Chhabra SK. Increased oxidative stress and altered levels of antioxidants in chronic obstructive pulmonary disease. Inflammation. (2005) 29:23–32. doi: 10.1007/s10753-006-8965-3 16502343

[34] Zhu N Lin S Yu H Liu F Huang W Cao C. Naples prognostic score as a novel prognostic prediction indicator in adult asthma patients: a population-based study. World Allergy Organ J. (2023) 16:100825. doi: 10.1016/j.waojou.2023.100825, PMID: 37954399 PMC10632111

[35] Wu WW. Association of Naples prognostic score and lung health: a population-based study. Respir Med. (2024) 232:107751. doi: 10.1016/j.rmed.2024.107751, PMID: 39089390

