# Supplementary figures and images for: Prognostic value of the Naples Prognostic Score in adult chronic obstructive pulmonary disease: NHANES 2005–2018
Source: Front Nutr. 2024 Dec 16;11:1502266. doi: 10.3389/fnut.2024.1502266 (PMC11682890; doi:10.3389/fnut.2024.1502266)

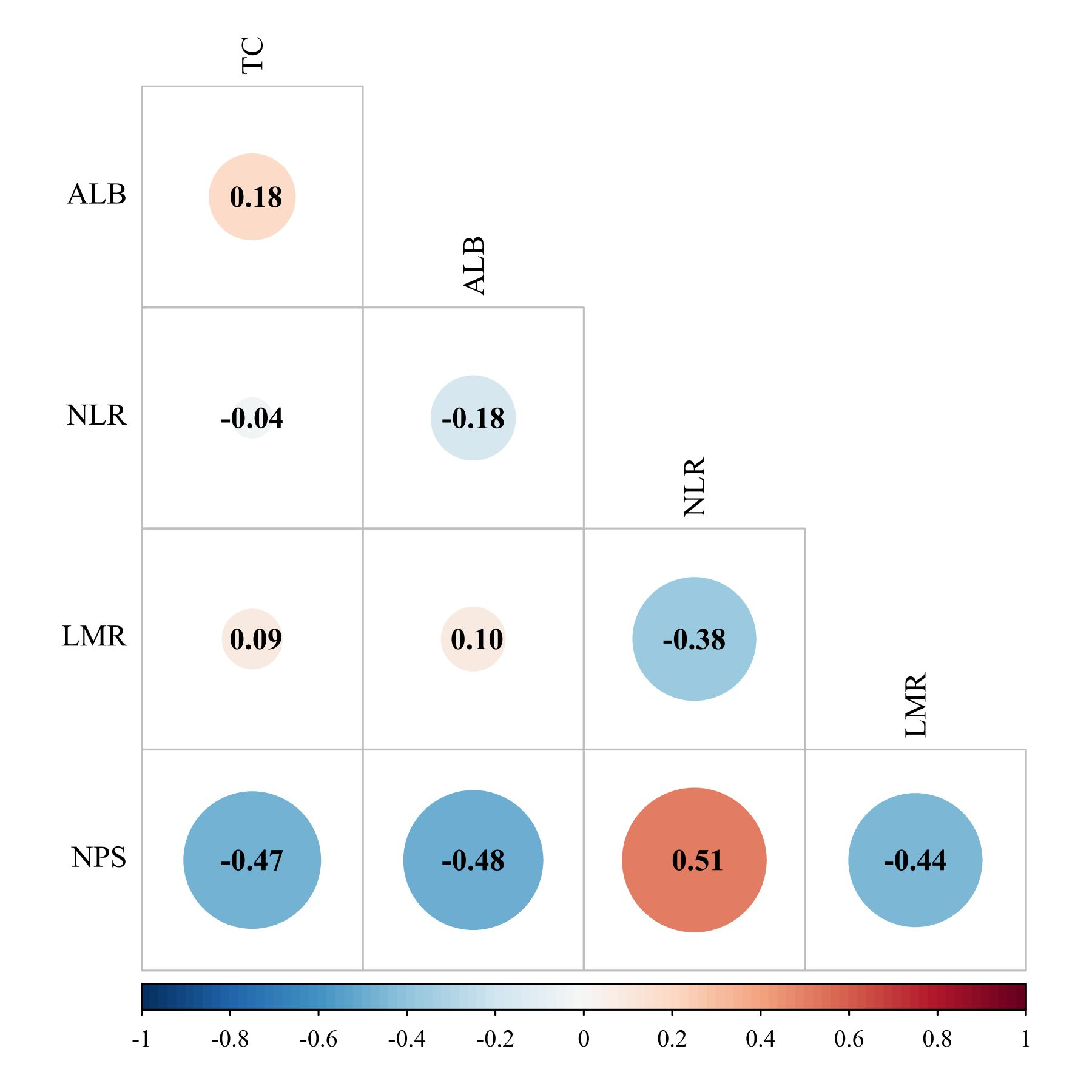

Supplement: APPENDIX FIGURE 1A — Spearman’s correlation analysis among NPS and its components. [file Image_1.TIFF]

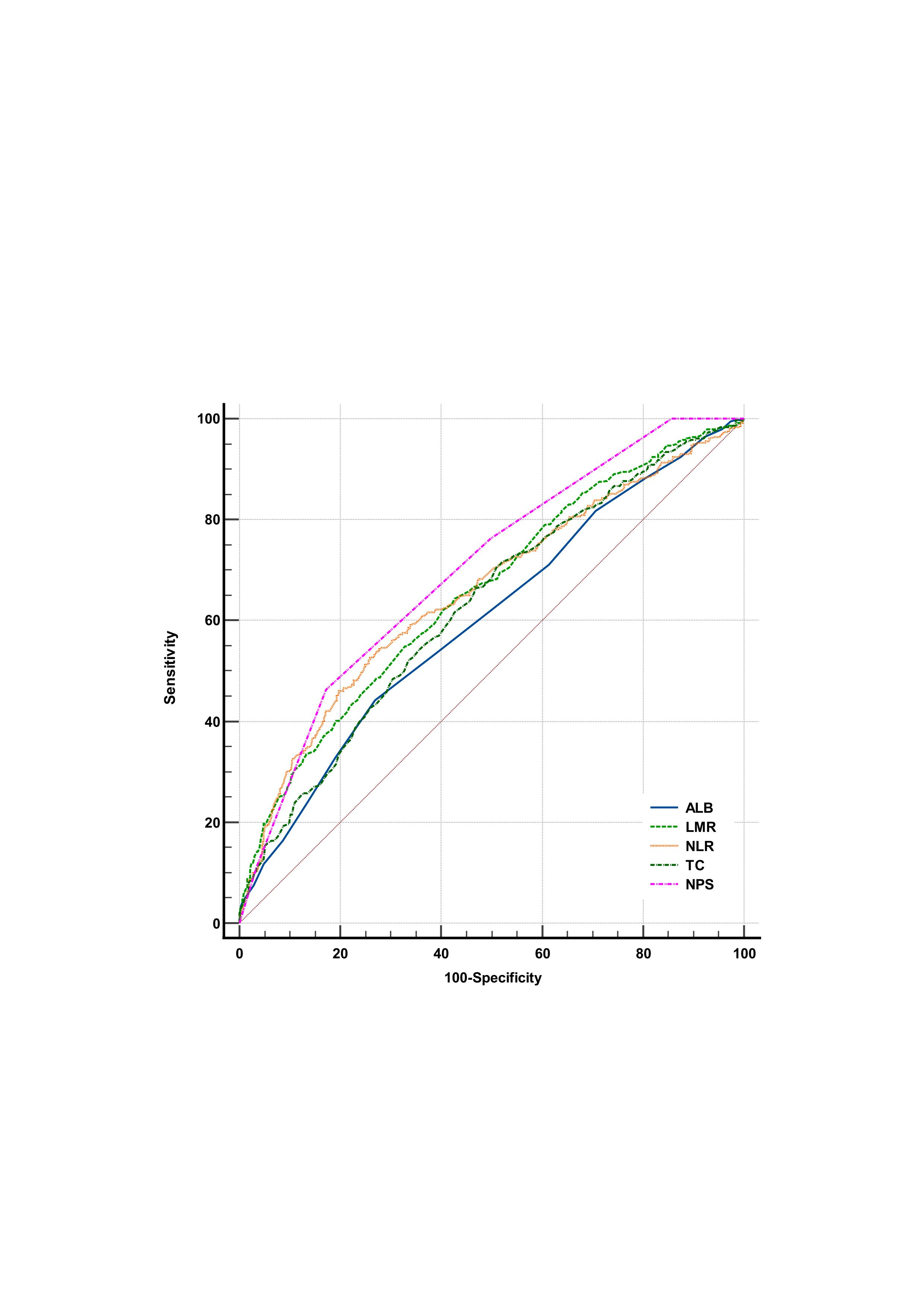

Supplement: APPENDIX FIGURE 1B — ROC curves of NPS and its components in predicting all-cause mortality in COPD patients. [file Image_2.TIFF]
