# Supplementary material for: Prognostic value of the Naples Prognostic Score in adult chronic obstructive pulmonary disease: NHANES 2005–2018
Source: Front Nutr. 2024 Dec 16;11:1502266. doi: 10.3389/fnut.2024.1502266 (PMC11682890; doi:10.3389/fnut.2024.1502266)
Supplement: Supplementary file 3 [file Data_Sheet_1.PDF]

Appendix Table 1. AUC of NPS and its components in predicting all-cause mortality in COPD patients in COPD patients in COPD patients

Parameters	AUC	SE [a]	95% CI [b]
ALB	0.602	0.0170	0.576 - 0.627
LMR	0.656	0.0164	0.631 - 0.680
NLR	0.656	0.0170	0.631 - 0.680
TC	0.627	0.0165	0.602 - 0.652
NPS	0.701	0.0144	0.676 - 0.724
